# Proximity to major roads and the incidence of osteoporotic fractures in elderly women: The BONE study in Beijing

**DOI:** 10.3389/fpubh.2022.1036534

**Published:** 2022-12-01

**Authors:** Yun Ren, Weishi Li, Zhongqiang Chen, Jue Liu, Dongwei Fan

**Affiliations:** ^1^Department of Obstetrics and Gynecology, Peking University Third Hospital, Beijing, China; ^2^Department of Orthopaedics, Peking University Third Hospital, Beijing, China; ^3^Beijing Key Laboratory of Spinal Disease Research, Beijing, China; ^4^Engineering Research Center of Bone and Joint Precision Medicine, Ministry of Education, Beijing, China; ^5^Department of Epidemiology and Biostatistics, School of Public Health, Peking University, Beijing, China

**Keywords:** air pollution, osteoporotic fracture, motor vehicle pollution, road, street

## Abstract

**Background:**

There is growing evidence to suggest that living near major roads (and suffering from the air pollution of urban streets) can have an adverse effect on bone health. However, little is known about its relationship to fractures caused by osteoporosis.

**Objective:**

This study was designed to investigate the relationship between residents living near major roads and the incidence of osteoporotic fractures.

**Methods:**

A retrospective cohort of 529 subjects was established based on community populations in older women aged 65–91. All participants lived in Beijing between September 27, 2007 and September 26, 2017. The distance between the residential sites of the subjects and the main roads was determined by the authors. Osteoporotic fracture diagnosis was based on medical histories and imaging examinations (DXA and X-rays). The Cox proportional hazard model was used to assess the association between traffic proximity and osteoporotic fractures, with suitable adjustments for individual and background factors.

**Results:**

The age range of all participants was 65–91 years, with an average age of 75.8 years (and a standard deviation 6.8 years). Of these, 19 (3.59%) suffered from diabetes, and 48 (9%) had hypertension; 85 (14%) families had annual incomes below US $30,000 and 402 (76%) had received a secondary school education or higher. Nearly 25% of people lived within 50 m of a main road, while 50% lived within 300 m. Between 2007 and 2017, a total of 96 osteoporotic fractures were observed. For people living <50 m from a main road, the adjusted hazard ratio (HR) for osteoporotic fractures was 2.509 (95% CI 1.345–4.680), while it was 1.830 (95% CI 1.029–3.255) for those living at a distance of 50–300 m from a main road vs. those living further than 300 m away.

**Conclusion:**

In this community-based cohort, living near a major road was associated with a higher incidence of osteoporotic fractures.

## Introduction

Osteoporosis is characterized by decreased bone mass, increased bone fragility and increased risk of fracture. Among osteoporosis patients over the age of 50, about one-third of women and one-tenth of men suffer from fragile fractures for the rest of their lives (1). Within 1 year of the onset of fragile fractures, the risk of death increases by 10 to 20% (2–4), and those who survived the fracture are more likely to suffer recurrent fractures, develop chronic pain, or endure a reduced quality of life (2, 5, 6). Each year, about 12.3 million people in the United States suffer from osteoporosis, which includes 2.1 million cases of fragility fractures, with direct medical costs as high as 20 billion US dollars (7). The prevalence of osteoporosis in people in China over the age of 50 is 19.2%, of which 6.0% are men and 32.1% are women. The prevalence of osteoporosis in people over 65 years of age reaches 32.0%, of which men account for 10.7% and women for 51.6%. By 2050, there will be 5.9 million osteoporotic fractures in China, and the resulting medical expenditure will be more than 20 billion US dollars (8, 9). In recent years, studies have shown that living near major roads is associated with reduced bone density and increased risk of osteoporosis, but its impact on osteoporotic fractures is still unclear.

Living near a city's major roads can significantly increase personal exposure to traffic-related air pollution (such as fine particulate matter, nitrogen oxides, polycyclic aromatic hydrocarbons, volatile organic compounds, heavy metals, and particles released from the wear of tires and friction materials, noise and other factors). As the world's urban population increases every year, more and more people are living closer to main urban roads. Therefore, the association between traffic exposure due to residential proximity to major roads and the incidence of osteoporotic fractures in an urban community-based cohort in Beijing, China were the main focus of study for this paper.

## Background and methodology

### Study design and participants

This study was part of the Beijing Osteoporosis with Neurological Disorders in Epigenetic Changes Study (BONE) (ClinicalTrials.gov ID: NCT03401619). Briefly, the BONE study is a community-based, ambispective cohort study of clinical and subclinical osteoporosis associated with neurological disorders. Between 2017 and 2018, 2,000 adults aged 55–102 from six communities in the Beijing area were enrolled in this study (this included the Mudanyuan, Jimenli, Xiaonanzhuang, Lanqiying, Jiuxianqiao, and Changchunjie communities). The study procedure and protocol were approved by the ethics committee of the Peking University Third Hospital at Peking University, and all work was performed in accordance with the Declaration of Helsinki. All participants provided written informed consent.

**Figure 1:**
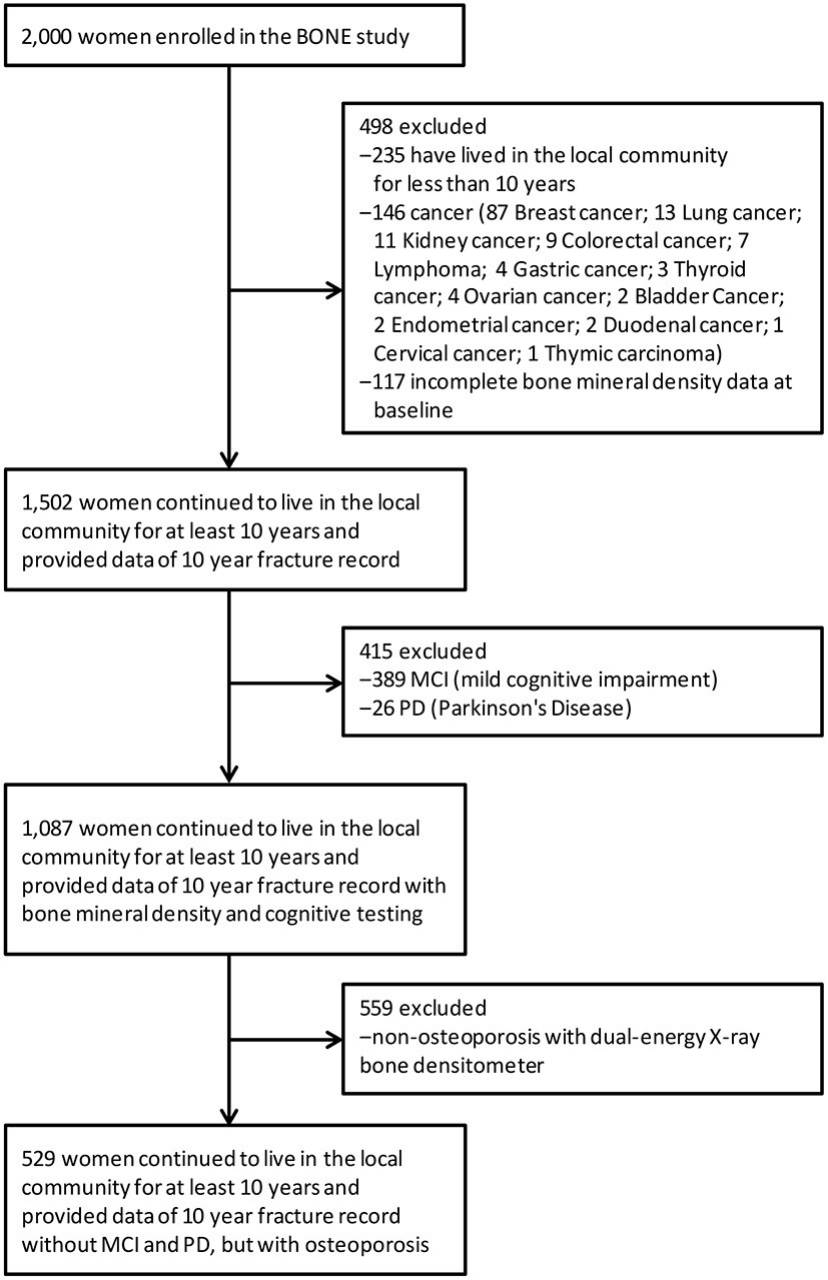
Participant flow.

For this study, participants were excluded if they had lived in the local community for <10 years (*N* = 235), or if they had cancer (*N* = 146) or incomplete bone mineral density data at baseline (*N* = 117), or had MCI (mild cognitive impairment) (*N* = 389), PD (Parkinson's Disease) (*N* = 26), or no evidence of osteoporosis (*N* = 559) (Figure 1). This left a final analytic sample of 529 participants, with data collected from September 2007 to September, 2017. The demographic characteristics of the subjects that were included in the study were similar to those of excluded subjects (Supplementary Table 1).

### Ascertainment of osteoporotic fractures

One key factor within this study was evidence of an osteoporotic fracture which was caused without trauma and with no more than mild violence. A mildly violent injury is defined as an injury caused by a fall on flat ground or an unintentional fall to the ground at a lower level, with no evidence of violence, loss of consciousness, hemiplegia, or falls as a result of seizures. If there is a sudden, involuntary change in posture, falling to the ground or to a lower plane can be included in the definition of mild violence. X-ray or CT examinations are used to diagnose the fracture standards. If a fracture was caused by a major trauma (such as a motor vehicle accident) or a bone-related disease, rather than by mild violence, it was excluded from the analysis. The most common sites of fragile fractures in the elderly are the spine, hip, distal radius and proximal humerus. In this study, the primary focus was on hip fractures, vertebral fractures and wrist fractures. The outcomes of the study were followed up, with relevant data collected, every 2 years.

### Residential proximity to roads

The distance to the main roads of the city were calculated based on the specific building codes where participants in the cohort lived within their community. The distance (m) was measured using ArcGIS. According to the Beijing Road Network, main traffic roads include urban main roads and arterial roads (that is, roads with medium-to-large traffic volumes and a combination of controllable traffic and peaceful intersections). Consistent with previous research (17, 24), three distance categories were created: <50 m from the main road, 50–300 m away, and more than 300 m away. Continuous distance measurement was also considered. In addition, in order to more accurately refine the exposure, it was also calculated whether the high-rise residential apartments on either side of the road were facing the road or facing away from it.

### Covariates

Data was collected related to each participant's general physical condition, as well as risk factors for osteoporotic fractures. These included age, weight, BMI, existing comorbidities (hypertension, hyperlipidemia, diabetes, coronary heart disease, stroke), level of education (uneducated or elementary school, high school or below, college and higher) and monthly household income (<CNY¥ 5,000, CNY¥ 5,000–19,999, ≥CNY¥ 20,000). Risk factors for osteoporotic fractures included: parental history of hip fracture, long-term glucocorticoid medication, history of rheumatoid arthritis, smoking, alcohol consumption [these indicators were taken from the osteoporotic FRAX fracture risk factor assessment tool recommended by the World Health Organization (WHO), which is used to calculate the probability that a subject will suffer a major fracture (hip, spine, humerus and wrist) in the next 10 years].

### Exposure assessment

In order to explore whether exposure to nitrogen dioxide (NO_2_), a typical urban street air pollutant, is associated with osteoporotic fractures, the land use regression method (LUR) was used to evaluate the long-term NO_2_ exposure of all participants. In brief: data was taken from 35 air quality monitoring sites on the website of the Beijing Environmental Monitoring Center (http://zx.bjmemc.com.cn/). These 35 air pollution monitoring points were divided into four categories: 12 urban environmental assessment points, 16 suburban environmental assessment stations, and 5 traffic pollution monitoring station tracking stations [the 5 traffic environment monitoring stations in Beijing are Yongdingmen Inner Street (Yongding Gate for short), Qianmen East Street (Qianmen for short), Xizhimen North Street (Xizhimen for short), South Third Ring Road West (South Third Ring for short), and East Fourth Ring Road North (East Fourth Ring for short) monitoring points]. Miyun, the reservoir station, served as a background reference point. The equipment used for vehicle flow collection consisted of a microwave multi-lane vehicle detection radar (RTMS) with a time resolution of 1 h and two regional background control stations. The predictor variables included the street network, land cover, population density, bus station density, and intersection density. An LUR model for air pollution was thus generated in GIS. The adjusted *R*^2^ size of the yearly LUR model was 0.74 to 0.86, suggesting that the model is suitable for explaining most of the variations. After applying a time adjustment, an estimate of LUR NO_2_ for each year between 2007 and 2017 could be obtained. Each participant's residence was then interpolated to obtain an estimate of their exposure to NO_2_.

### Physical activity assessment

After verification of reliability and validity, the Chinese version of the Physical Activity Scale for the Elderly (PASE) was used to measure and evaluate the physical activity levels of participants over the past week (10, 11). This includes physical exercise, housework and occupational physical activity. Physical exercise includes walking, low, medium and high intensity physical exercise, strength and endurance exercise activities. Housework includes housekeeping, cleaning, and household repairs. Occupational physical activities cover physical activity engaged in during employment and volunteer work. After completing the questionnaire, scores for various physical activities were calculated by means of different test question score weighting, before all activities were summarized to calculate the PASE score. PASE measurements are divided into low (0–99), medium (100–249) and high (≥250).

### Diet assessment

Standard diet was assessed by means of a validated semi-quantitative Food Frequency Questionnaire (FFQ) survey for China (12, 13). The consumption of rice, wheat, meat, eggs, milk, vegetables, fruits, beans and other grains were estimated by category. Amounts of edible oil were estimated per family, and then divided to obtain values for consumption per family member. Participants were asked how often they consumed each food or food group in the past 12 months (daily, weekly, monthly, yearly, or never) and how much of each they consumed (50 g /liang). The amount of food consumed by individuals was estimated based on the food type or category, such as the amount of wheat flour per meal, for example: <50, 50–100, 150–200, 250–300, 350–400, or 500 or more. Egg intake per meal was divided into 1 egg, 2 eggs, 3 eggs, 4 eggs, or 5 or more eggs. Based on the food consumption data and the Chinese Food Composition, an individual subject's nutrient intake could be calculated. FFQs were validated against multiple 24-h diet recalls. These recalls were made among randomly selected cohort participants (14, 15). The FFQ shows quite high effectiveness and repeatability.

### Quality control

The investigators were the members of the research team, all of whom were employed PhD students at Peking University. All investigators underwent specialist training and passed the necessary assessments to formally carry out this investigation. The questionnaire survey used a one-on-one, face-to-face survey method, with quality control personnel arranged to supervise and guide the survey site. After the daily survey had been completed, the quality control personnel would review the questionnaires to look for any missing items or logical errors; this could also involve calling the research subject to supplement or correct any of the information provided. The research project team held group meetings once a week to summarize and discuss the problems encountered during the survey process in the previous week. In addition, the project team would arrange for a regular assessment of the investigators.

### Statistical analysis

Continuous variables are described as mean (standard deviation), while categorical variables are described as frequency (percentage). Student's *t*-test was used to evaluate the difference of continuous variables, while Pearson's χ2 test or Fisher's exact test were used to evaluate the difference in categorical variables. Cox proportional hazards models were used, with age as the timescale, in order to assess the relationship between residential proximity to major roadways and incidence of osteoporotic fractures. For each outcome, the follow up time (in days) was measured from Sept 27, 2007 until the osteoporotic fracture date, or until Sept 26, 2017. Separate models were developed for osteoporotic fracture. All models were adjusted for age, economic status, education, physical activity, diet, smoking, alcohol intake, comorbidities and risk factors for osteoporotic fractures. The two co-authors (Yun Ren and Dongwei Fan) carried out statistical analysis and cross-checks to ensure the quality of the research. SPSS (version 17.0; IBM, Inc) was used for all statistical analysis. Two-sided *P*-values of <0.05 were considered statistically significant.

## Results

### Characteristics of subjects

Participant baseline characteristics included 529 women, all of whom were over 65 years old at the time of enrollment. The age range was 65–91 years, with an average age of 75.8 years (standard deviation 6.8 years). 19 (3.59%) had diabetes, and 48 (9%) had hypertension; the families of 85 (14%) participants had annual incomes below US $ 30,000, while 402 subjects (76%) had a secondary school education or above.

Nearly 25% of people lived within 50 m of a main road, while 50% lived within 300 m. Within the cohort, the average concentration of NO_2_ exposure between 2007 and 2017 was 53.8 μg/m^3^ (range: 34 to 87 μg/m^3^) based on the location of the participants' residences. Between 2007 and 2017, 96 cases of osteoporotic fracture were identified. Most fractures occurred in the vertebrae (62%), hips (27%) and wrists (11%). Demographics, physical, and clinical characteristics at baseline in the BONE study cohort can be seen in Table 1.

**Table 1:** Demographic, physical, and clinical characteristics at baseline in the BONE study cohort.

	**Total**	**Non-fracture**	**Fracture**	***p***
Age, mean (SD)	75.76 ± 6.80	75.65 ± 6.82	76.25 ± 6.71	0.434
Height, mean (SD)	158.30 ± 7.74	158.54 ± 7.93	157.25 ± 6.81	0.106
Weight, mean (SD)	59.68 ± 6.17	59.56 ± 6.21	60.20 ± 5.97	0.363
BMI, mean (SD)	23.98 ± 3.41	23.88 ± 3.52	24.44 ± 2.87	0.098
Menopause, mean (SD)	49.80 ± 3.80	50.00 ± 3.86	48.90 ± 3.38	0.010
NO_2_ (ug/m^3^), mean (SD)	53.80 ± 8.71	53.02 ± 8.15	57.32 ± 10.21	<0.001
**Education degree**, ***n*** **(%)**				0.314
No formal education or Elementary school	344(65.03)	287(66.28)	57(59.38)	
Less than or equal to high school	137(25.90)	110(25.40)	27(28.13)	
College and higher	48(9.07)	36(8.31)	12(12.50)	
**Monthly household income**, ***n*** **(%)**				0.008
<CNY¥ 5,000	98(18.53)	72(16.63)	26(27.08)	
CNY¥ 5,000–19,999	352(66.54)	301(69.52)	51(53.13)	
≥CNY¥ 20,000	79(14.93)	60(13.86)	19(19.79)	
**PASE level**, ***n*** **(%)**				0.825
Low (0–99 units)	71(13.42)	59(13.63)	12(12.50)	
Middle (100–200 units)	395(74.67)	321(74.13)	74(77.08)	
High (>200 units)	63(11.91)	53(12.24)	10(10.42)	
**Hypertension**, ***n*** **(%)**				0.167
No	469(88.66)	380(87.76)	89(92.71)	
Yes	60(11.34)	53(12.24)	7(7.29)	
**Hyperlipidemia**, ***n*** **(%)**				0.207
No	490(92.63)	404(93.30)	86(89.58)	
Yes	39(7.37)	29(6.70)	10(10.42)	
**Diabetes**, ***n*** **(%)**				0.975
No	510(96.41)	418(96.54)	92(95.83)	
Yes	19(3.59)	15(3.46)	4(4.17)	
**Coronary heart disease**, ***n*** **(%)**				0.500
No	515(97.35)	423(97.69)	92(95.83)	
Yes	14(2.65)	10(2.31)	4(4.17)	
**Stroke**, ***n*** **(%)**				0.807
No	517(97.73)	424(97.92)	93(96.88)	
Yes	12(2.27)	9(2.08)	3(3.13)	
**Parental history of hip fracture**, ***n*** **(%)**			<0.001
No	503(95.09)	420(97.00)	83(86.46)	
Yes	26(4.91)	13(3.00)	13(13.54)	
**Glucocorticoid**, ***n*** **(%)**				0.406
No	516(97.54)	424(97.92)	92(95.83)	
Yes	13(2.46)	9(2.08)	4(4.17)	
**Rheumatoid arthritis**, ***n*** **(%)**				0.406
No	516(97.54)	424(97.92)	92(95.83)	
Yes	13(2.46)	9(2.08)	4(4.17)	
**Smoking**, ***n*** **(%)**				0.164
No	489(92.44)	397(91.69)	92(95.83)	
Yes	40(7.56)	36(8.31)	4(4.17)	
**Alcohol**, ***n*** **(%)**				0.002
No	438(82.80)	348(80.37)	90(93.75)	
Yes	91(17.20)	85(19.63)	6(6.25)	

### Correlation and multiple linear regression analysis

**Figure 2:**
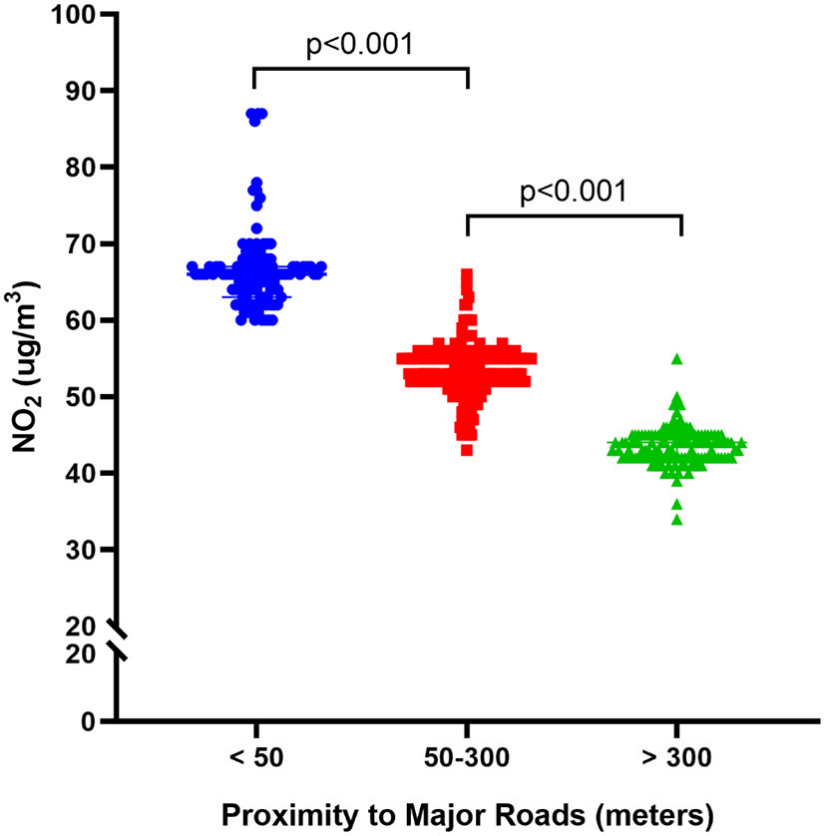
Long-term exposure to NO2 and risk of the residential proximity to roads for osteoporotic fractures.

Among the three groups, the difference in NO_2_ exposure levels was statistically significant. The NO_2_ level at <50 m was 66.47±5.22, while at 50–300 m it was 53.68 ± 3.33, and at >300 m it was 43.50 ± 2.43. As the distance from the main road increased, the NO_2_ levels decreased, with the difference between the three groups considered statistically significant (*p* < 0.001, Figure 2).

**Figure 3:**
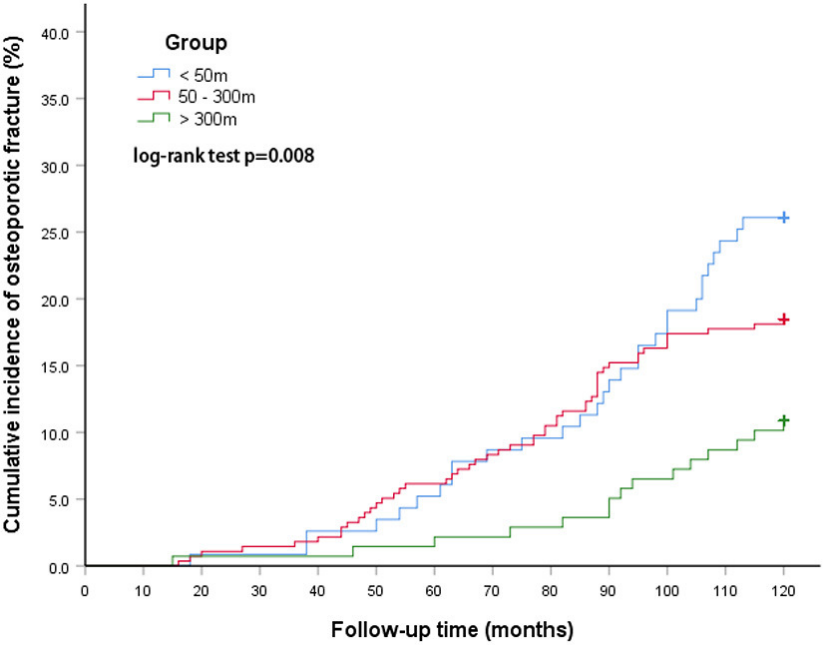
The cumulative incidence of osteoporotic fractures between groups at different distances from the main road.

There was also a statistically significant difference in the incidence of osteoporotic fractures between groups living at different distances from the main road. The cumulative incidence at <50 m was 26.1%, going down to 18.5% for 50–300 m, and 10.9% for >300 m. The log-rank test showed that the difference between the three groups was therefore statistically significant (*p* = 0.008, Figure 3).

### COX regression analysis

**Figure 4:**
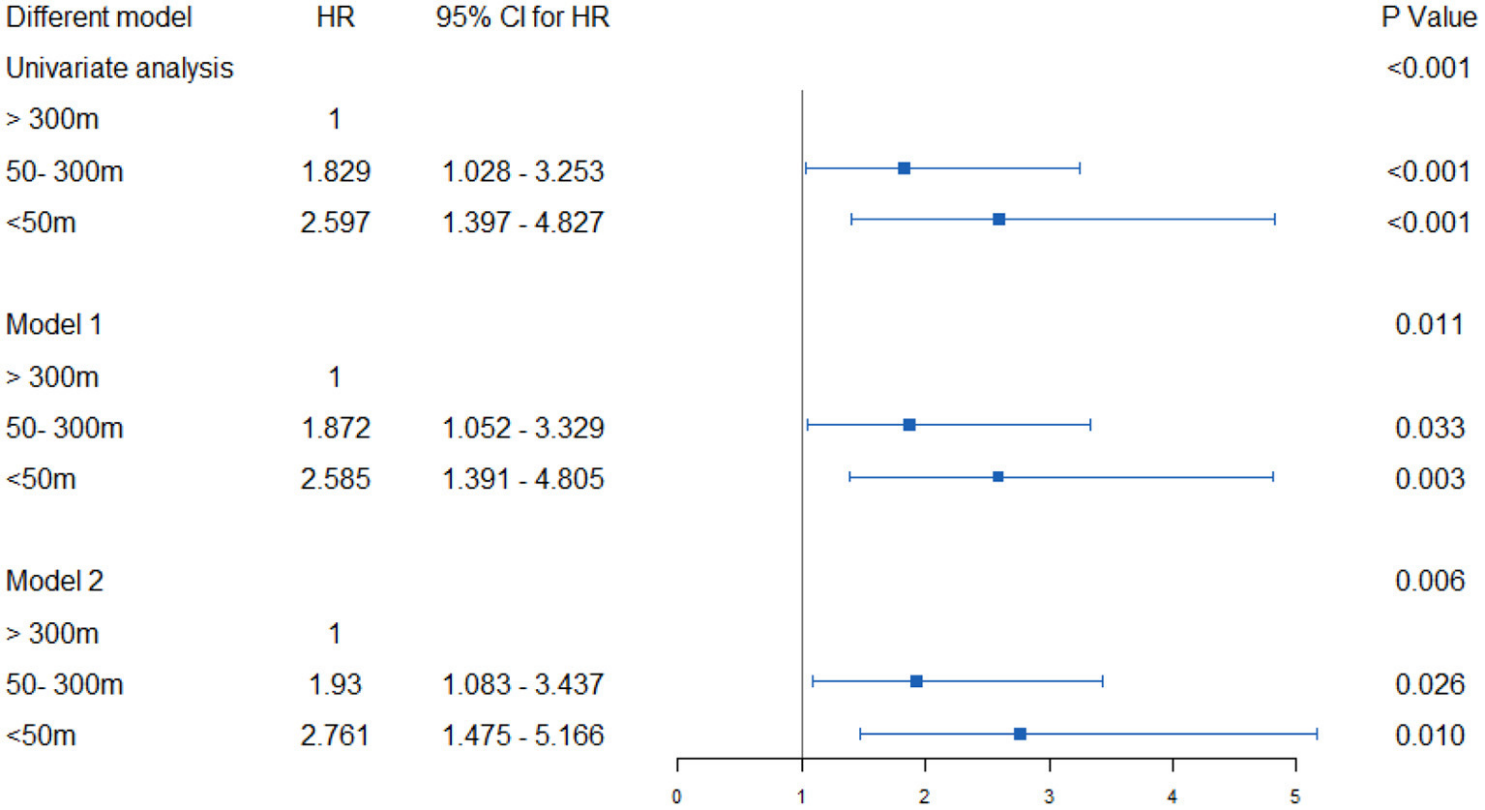
Hazard ratios and 95% CI for associations between residential proximity to major roadways in 2007 and the risk of incident osteoporotic fracture in Beijing during the follow-up period 2007–17. Indirect adjustment for smoking, body-mass index (BMI), physical activity, and attained education. Data of smoking, BMI, physical activity, and educational attainment were obtained from BONE study, and who were 55 to 81 years old at the time of the surveys (*n* = 529). Major traffic roads include primary urban roads and arterial roads, as defined by Beijing Government Road Network Data Standards. Cox proportional hazards model with age as time axis, adjusted for history of diabetes, hypertension, coronary heart disease, stroke, household income, education, etc. Using the natural logarithm of distance, set the distance as a continuous variable. The hazard ratio represents the distance that increases every intertertrtile-range. model 1: Age, BMI, Menopause, education degree, monthly household income, PASE level; Model 2: ge, BMI, Menopause, education degree, monthly household income, PASE level, hypertension, hyperlipidemia, diabetes, coronary heart disease, stroke, parental history of hip fracture, glucocorticoid rheumatoid arthritis, smoking, alcohol.

When conducting univariate COX regression analysis, proximity to the main road was related to an increased incidence of osteoporotic fractures. For people within 50 m, the cruded HR was 2.597 (95% CI 1.397–4.827), going down to 1.829 (95% CI 1.028–3.235) for those living at a distance of 50–300 m from the road, vs. those living further than 300 m. For this form of analysis, after considering the confounding factors of age, BMI, menopausal age, degree of education, monthly household income, and PASE level, the adjusted HR between those living within 50 m and 50–300 m, respectively were 2.585 (1.391–4.805) and 1.872 (1.052–3.329), when compared to those living further than 300 m away. After being adjusted for other confounding factors, similarly adjusted HRs were obtained, with the results remaining stable. This shows that people living near major roads have a higher risk of osteoporotic fracture (Figure 4).

## Discussion

In this longitudinal cohort based on a community population, living near major roads was associated with an increased incidence of osteoporotic fractures. In dwellings located <50 m away from the main road, the HRs were 2.597 (95% CI 1.397 – 4.827), significantly higher than for people living at a distance between 50 and 300 meters from main roads. This means that the closer you are to the road, the greater the exposure to air pollution caused by traffic, and the higher the incidence of osteoporotic fractures. After correction for various risk factors, this association still exists.

As far as the researchers are aware, this is the first study of osteoporotic fractures related to traffic pollution. Living near major roads will greatly increase a person's exposure to traffic-related air pollution (for example, fine particulate matter, nitrogen oxides, heavy metals, polycyclic aromatic hydrocarbons, and particles produced by tire and friction material wear) and noise. Although the mechanism by which traffic exposure may affect bone health is not yet fully understood, traffic-related air pollution affects different organs and tissues, and obviously also affects bone health. PM2.5 is composed of particles with a diameter of <2.5 microns that can penetrate the alveolar system and directly enter the blood stream, becoming deposited in the deeper organs of the human body. This can trigger inflammation, affect bone metabolism, lead to bone loss, and endanger bone health (16–18). In addition to PM2.5, traffic pollutants such as polycyclic aromatic hydrocarbons and nitrogen oxides can also induce an immune reaction (19). We know that the skeletal system and the immune system are closely related in terms of anatomical position and function. Various immune cells develop from pluripotent bone marrow stem cells. T lymphocytes, B lymphocytes, cytokines, chemokines, and costimulatory molecules can all interact with osteoblasts and osteoclasts to jointly regulate bone formation and bone resorption, thereby changing the remodeling of bones. In patients with inflammatory and immune diseases, the prevalence of osteoporosis has been found to increase (20). Therefore, the association between traffic pollution exposure and lower BMD (Bone Mineral Density, BMD) is likely to be mediated by the activated immune system. Decreased vitamin D will result in a decrease in serum calcium levels, which will in turn lead to increased osteoclast activity and calcium migration from the bones to the extracellular space (21, 22). A study of 35 6-year-old children found that exposure to IL-6, which is found in higher concentration in outdoor traffic pollution, is closely related to vitamin D deficiency (23). Finally, living in areas of heavy traffic is also related to a reduction in outdoor activities (24, 25), This can reduce a person's exposure to the ultraviolet B (UVB) rays from the sun. Insufficient absorption of UVB by the skin is one of the main causes of vitamin D deficiency. In addition, air pollution causes an oxidative stress response in the body, as well as leading to endocrine disorders and other issues, which may act together to harm bone health.

Recent studies have shown that air pollution can damage bones and reduce bone density, and that it can also be associated with fractures. The Medicare study, conducted by Prada et al. (26), analyzed more than 9.2 million medical insurance beneficiaries, and found that there is an association between PM2.5 concentration and hospitalization rate for fractures, regardless of gender and community-level confounding factors. Chen et al. (27) analyzed cohort data for overweight and obese people, and found that among Mexican Americans living near major roads, their adult body and pelvic bone density levels were found to be lower; their research showed this was related to weight, height, and body fat percentage, but that physical activity, environmental variables, and daily calcium and vitamin D intake were irrelevant. Ultimately, traffic air pollution was found to reduce BMD and increase the risk of osteoporosis in Mexican Americans. Chang et al. (28) found an association between air pollution (carbon monoxide and nitrogen dioxide) and increased risk of osteoporosis. After men and women are exposed to NO_2_, the correlation between this exposure and the risk of osteoporosis increases. The research carried out in this paper observed that NO_2_ exposure is also closely related to osteoporotic fractures.

However, the fact that NO_2_ cannot fully explain the effect of osteoporotic fractures suggests that other pollutants, possibly PM2.5 or other factors (such as heavy metals, polycyclic aromatic hydrocarbons, noise) may have played a role. Prada et al. (26) reported that, for every 4.18 μg/m3 increase in PM2.5, the fracture admission rate in elderly people increased by 4.1%. Among these, it was observed that PM2.5 levels could be correlated with rates of hospital admission for elderly people over 65 years of age due to fractures. Ranzani et al. (29) conducted a population-based cross-sectional analysis of a cohort of parents and children in Andhra Pradesh. The study recruited participants from 28 villages near Hyderabad in southern India between 2009 and 2012. It was shown that for people in the southern suburbs of the city, air pollution led to reduced BMD. Alvaer et al. (30) conducted a cohort study in Oslo. The subjects of the study were men and women aged 59/60 and 75/76, respectively. The individualized air pollution indicators were compared with forearm BMD and forearm fractures. The results found that every increase of one standard deviation of PM2.5 (μg/m^3^) reduced the average BMD of the distal forearm by 19 mg/cm^2^. Since PM2.5 measurements were not carried out in Beijing until 2013, and since the starting point for the cohort in this study was 2007, PM2.5 was not included in the analysis since 6 years' worth of data was missing. However, Beijing's layout of air quality monitoring stations in adjacent streets gives an average value of about 73 μg/m^3^ in the 5 years from 2013 to 2017, which is much higher than that of non-adjacent streets. In addition, PM2·5 concentrations from 2013 to 2017 in adjacent streets in Beijing were much higher than in the northeast-mid-Atlantic USA. The results therefore suggest that PM2.5 may also be one of the reasons for the higher incidence of osteoporotic fractures in people living close to main roads.

Heavy metal pollution is a major component of urban air pollution, and an important cause of damage to bone health (31, 32). Wong et al. (33) performed X-ray fluorescence spectroscopy and mass spectrometry on 38 postmenopausal women in the Hamilton Cohort of the Canadian Multicenter Osteoporosis Study (CaMos) to detect lead content in bones and blood, and found that lead accumulated in bones not only caused damage to bone structure and bone density, but also reduced the number of bone trabeculae, increased the distance between trabecular bones, and reduced bone density. At the same time, it also caused the concentration of lead in the blood to remain high, leading to associated organ damage. Theppeang et al. (34) tested 112, 50–70-year-old women living in Baltimore, Maryland, USA, including whites and African Americans, for bone and blood lead levels, and their associated effects on bone health; it was found that the lead accumulated in the bones is harmful to human health. In addition to lead exposure, chronic cadmium exposure is also closely related to osteoporosis (35), and can even affect bone development in children. Malin et al. (36) tested 504 children in a mother-infant cohort in Bangladesh using blood and urine cadmium concentration levels and biomarkers for bone remodeling at the ages of 9 and 4.5, as well as during pregnancy. It was found that cadmium exposure in children caused abnormal bone development, which was negatively correlated with abnormal bone metabolism indicators such as vitamin D3 (37). Further studies on the mechanism of chronic cadmium (Cd) poisoning leading to damaged bones found that chronic cadmium poisoning can cause the down-regulation of mesenchymal stem cell osteogenic differentiation genes, such as Osterix, Osteopontin, Type I collagen, Type 2 collagen and Osteogenesis Transcription Factor 2 (RUNX2). In particular, cadmium can directly act on MSCs through the classical bone metabolism molecular signaling pathway RANKL/OPG pathway, leading to abnormal bone formation (38). Cong et al. (39) collected 36 road dust samples from Beijing and analyzed these for heavy metals (As, Cd, Cr, Cu, Hg, Mn, Ni, Pb, Zn, Fe). Except for arsenic and manganese, most of the metals exceed their corresponding background values, with the average concentration of cadmium in particular found to be eight times the background value. This data indicates that heavy metal pollution in the air on urban streets may also be an important cause of osteoporotic fractures in elderly women.

In addition, polycyclic aromatic hydrocarbon (PAH) pollution, automobile exhaust (40), coke smelting exhaust gas (41), cigarette smoke (42), and polycyclic aromatic hydrocarbons (PAH) are closely related to bone health. The chemical substances most closely associated with BMD are polycyclic aromatic hydrocarbons (PAH). Cohort studies found that PAH is closely related to osteoporosis (43, 44). Further animal studies have also shown that PAHs may increase osteoclast metabolism and reduce osteogenic capacity, leading to a loss of bone mass and strength (45). These pollutants in the air on urban streets may also be the causative factors of osteoporotic fractures in elderly women.

The real-life air pollution situation is a little complicated. Stimuli act on organs and tissues (46), leading to changes in intracellular signaling pathways. For example, these heavy metal issues can change the WNT signaling pathway that is key to the osteogenesis process (47), affecting normal bone metabolism and leading to heavy metal exposure. The bone microstructure is subsequently destroyed, leading to bone injury (48). There are also articles researching the use of cell and animal experiments to explore the application of melatonin (35), Aronia black fruit polyphenols (49), and other methods to reduce bone damage caused by exposure to heavy metals such as cadmium. Of course, these articles also suggest that these methods are merely remedies, the most fundamental and urgent measure being the need to control the original sources of air pollution.

There were certain limitations in this study. Firstly, changes in building and road structures were not examined during the studied period. In spite of this fact, people who participated in the cohort had lived in the corresponding community for a long time, and the surrounding buildings and roads did not change significantly during this period. Secondly, only behavioral information was collected (such as smoking and alcohol use) at the baseline, meaning that changes in behavioral habits could not be controlled within the models. However, given that elderly women in China commonly show relatively low and relatively consistent rates of smoking and alcohol consumption, these changes in behavioral variables were felt to be of limited impact on the overall results. Thirdly, NO_2_ exposure levels were calculated based on the participants' home address, which only considers the exposure level when living at home, and did not estimate other types of exposure, such as NO_2_ exposure at work, which may have led to certain measurement errors.

Recent studies have shown that characterizing these mechanisms will better determine the physiopathology of bone injuries, while recognizing the fact that air pollution is a modifiable risk factor for osteoporosis will provide a basis for environmental policy. This knowledge will also be useful in guiding the prevention of fractures due to fragility and help reduce health-related costs. Air pollution in general, and street air pollution in particular, has important direct social and clinical consequences for the prevention of osteoporotic fractures. Any policy aimed at reducing particulate emissions and their gaseous precursors can help prevent osteoporotic fractures and associated complications. It is especially meaningful for highly industrialized areas with serious levels of air pollution, because in these areas, emissions are still increasing rapidly, while the population is aging faster than ever before.

To sum up, there is a positive correlation between traffic vehicle pollution and osteoporotic fractures in Beijing's urban streets, and increased air pollution will lead to further incidence and increased severity of osteoporotic fractures. Therefore, air pollution caused by urban motor vehicles should be a key area of focus, and all levels of government need to strengthen their overall control of air pollution. Furthermore, in this age of serious pollution, a better job needs to be done in the prevention and treatment of osteoporotic fractures in old age, in order to reduce their occurrence and to reduce the incidence of morbidity.

## Data availability statement

The original contributions presented in the study are included in the article/Supplementary material, further inquiries can be directed to the corresponding author.

## Ethics statement

The studies involving human participants were reviewed and approved by the Ethics Committee of Peking University Third Hospital, Peking University. The patients/participants provided their written informed consent to participate in this study.

## Author contributions

YR analyzed the data, wrote the first draft of manuscript, and obtained the data. JL and DF conceptualized the manuscript and participated in study design. WL assisted with the initial data analyses. JL, DF, and WL reviewed the draft of manuscript. ZC helped to revise the article. All authors have read and approved the manuscript.

## Funding

This work was supported by grants from National Natural Science Foundation of China (42277420, 30801156, and 41931291), National Key Research and Development Program of China (2017YFC0113001), Program for Training Capital Science and Technology Leading Talents (Z181100006318003), and Fostering Young Scholars of Peking University Health Science Center (BMU2017PY015).

## Conflict of interest

The authors declare that the research was conducted in the absence of any commercial or financial relationships that could be construed as a potential conflict of interest.

## Publisher's note

All claims expressed in this article are solely those of the authors and do not necessarily represent those of their affiliated organizations, or those of the publisher, the editors and the reviewers. Any product that may be evaluated in this article, or claim that may be made by its manufacturer, is not guaranteed or endorsed by the publisher.
