# Supplementary material for: Proximity to major roads and the incidence of osteoporotic fractures in elderly women: The BONE study in Beijing
Source: Front Public Health. 2022 Dec 1;10:1036534. doi: 10.3389/fpubh.2022.1036534 (PMC9752863; doi:10.3389/fpubh.2022.1036534)
Supplement: Supplementary file 1 [file Table_1.DOCX]

**Supplemental table 1. Comparison of demographic characteristics between the subjects included and excluded**

|  | Included subjects | Excluded subjects | p |
| --- | --- | --- | --- |
| Age, mean (SD) | 75.76±6.80 | 75.79±6.74 | 0.944 |
| Education degree, n (%) |  |  | 0.638 |
| No formal education or Elementary school | 344(65.03) | 362(64.76) |  |
| High school or equal to high school | 137(25.90) | 154(27.55) |  |
| College and higher | 48(9.07) | 43(7.69) |  |
| Monthly household income, n (%) |  |  | 0.129 |
| <CNY¥ 5000 | 98(18.53) | 131(23.43) |  |
| CNY¥ 5000–19999 | 352(66.54) | 354(63.33) |  |
| ≥CNY¥ 20000 | 79(14.93) | 74(13.24) |  |
| Hypertension, n (%) |  |  | 0.360 |
| no | 469(88.66) | 489(87.48) |  |
| yes | 60(11.34) | 70(12.52) |  |
| Hyperlipidemia, n (%) |  |  | 0.010 |
| no | 490(92.63) | 492(88.01) |  |
| yes | 39(7.37) | 67(11.99) |  |
| Diabetes, n (%) |  |  | 0.124 |
| no | 510(96.41) | 528(94.45) |  |
| yes | 19(3.59) | 31(5.55) |  |
| Coronary heart disease, n (%) |  |  | 0.105 |
| no | 515(97.35) | 534(95.53) |  |
| yes | 14(2.65) | 25(4.47) |  |
| Stroke, n (%) |  |  | 0.201 |
| no | 517(97.73) | 539(96.42) |  |
| yes | 12(2.27) | 20(3.58) |  |
| Parental history of hip fracture, n (%) | |  | 0.229 |
| no | 503(95.09) | 522(93.38) |  |
| yes | 26(4.91) | 37(6.62) |  |
| Glucocorticoid, n (%) |  |  | 0.281 |
| no | 516(97.54) | 539(96.42) |  |
| yes | 13(2.46) | 20(3.58) |  |
| Rheumatoid arthritis, n (%) |  |  | 0.281 |
| no | 516(97.54) | 539(96.42) |  |
| yes | 13(2.46) | 20(3.58) |  |
| Smoking, n (%) |  |  | 0.057 |
| no | 489(92.44) | 498(89.09) |  |
| yes | 40(7.56) | 61(10.92) |  |
| Alcohol, n (%) |  |  | 0.178 |
| no | 438(82.80) | 445(79.61) |  |
| yes | 91(17.20) | 114(20.39) |  |

Notes: Comparison of demographic characteristics between the subjects 529 included and 559 excluded among the 1087 women continued to live in the local community for at least 10 years.
